# Chromosome-scale genome assemblies of wild tomato relatives *Solanum habrochaites* and *Solanum galapagense* reveal structural variants associated with stress tolerance and terpene biosynthesis

**DOI:** 10.1093/hr/uhac139

**Published:** 2022-06-20

**Authors:** Xiaofen Yu, Minghao Qu, Yanna Shi, Chenlu Hao, Sumin Guo, Zhangjun Fei, Lei Gao

**Affiliations:** CAS Key Laboratory of Plant Germplasm Enhancement and Specialty Agriculture, Wuhan Botanical Garden, Innovative Academy of Seed Design, Chinese Academy of Sciences, Wuhan 430074, China; Hubei Hongshan Laboratory, Wuhan 430070, China; CAS Key Laboratory of Plant Germplasm Enhancement and Specialty Agriculture, Wuhan Botanical Garden, Innovative Academy of Seed Design, Chinese Academy of Sciences, Wuhan 430074, China; University of Chinese Academy of Sciences, Beijing 100049, China; Zhejiang Provincial Key Laboratory of Horticultural Plant Integrative Biology, Zhejiang University, Hangzhou 310058, China; CAS Key Laboratory of Plant Germplasm Enhancement and Specialty Agriculture, Wuhan Botanical Garden, Innovative Academy of Seed Design, Chinese Academy of Sciences, Wuhan 430074, China; University of Chinese Academy of Sciences, Beijing 100049, China; CAS Key Laboratory of Plant Germplasm Enhancement and Specialty Agriculture, Wuhan Botanical Garden, Innovative Academy of Seed Design, Chinese Academy of Sciences, Wuhan 430074, China; Boyce Thompson Institute, Cornell University, Ithaca, NY 14853, USA; US Department of Agriculture-Agricultural Research Service, Robert W. Holley Center for Agriculture and Health, Ithaca, NY 14853, USA; CAS Key Laboratory of Plant Germplasm Enhancement and Specialty Agriculture, Wuhan Botanical Garden, Innovative Academy of Seed Design, Chinese Academy of Sciences, Wuhan 430074, China; Hubei Hongshan Laboratory, Wuhan 430070, China

##  

Dear Editor,

Introducing beneficial genes/alleles from wild relatives into the cultivated tomato has been an important approach for tomato breeding. *Solanum habrochaites* and *S. galapagense* have been widely used as germplasm donors in modern breeding to improve biotic and abiotic stress tolerance of tomato. *S. habrochaites* grows in the Peruvian Andes at altitudes up to 3300 m and is notable for its tolerance of chilling and drought and resistance to many diseases and pests. *S. galapagense* is endemic to the Galápagos Islands, has extraordinary salt tolerance and insect resistance, and appears even more closely related to the cultivated tomato (*Solanum lycopersicum*) than *Solanum pimpinellifolium*, the wild progenitor of cultivated tomato [1]. Due to their importance, draft genomes of these two species have been assembled using Illumina short-read sequencing [2] or PacBio long-read sequencing [3]. However, high levels of fragmentation and/or the lack of chromosome-scale assemblies have limited their applications in tomato breeding and research.

In this study, chromosome-scale assemblies of *S. habrochaites* (accession LA0407) and *S. galapagense* (accession LA0317) were developed using PacBio HiFi reads and chromatin interaction maps generated with Hi-C technology. The final assemblies of *S. habrochaites* and *S. galapagense* had total contig sizes of 950.7 and 859.9 Mb, respectively, and contig N50 sizes of 6.74 and 12.32 Mb, with 95.4 and 94.4% of the contigs anchored and ordered on the 12 chromosomes (Fig. 1A, Supplementary Data Fig. S1). The *S. habrochaites* and *S. galapagense* assemblies captured 97.6 and 98.5% of the 1614 Embryophyta conserved genes, respectively, and had LTR (long terminal repeat) assembly index (LAI) scores of 13.50 and 13.35. Moreover, the consensus quality values (QVs) of *S. habrochaites* and *S. galapagense* assemblies were 42.91 and 44.28, respectively, corresponding to a base accuracy of 99.995 and 99.996%. Taken together, the results indicated the high degree of contiguity, completeness, and base accuracy of these two genome assemblies.

**Figure 1:**
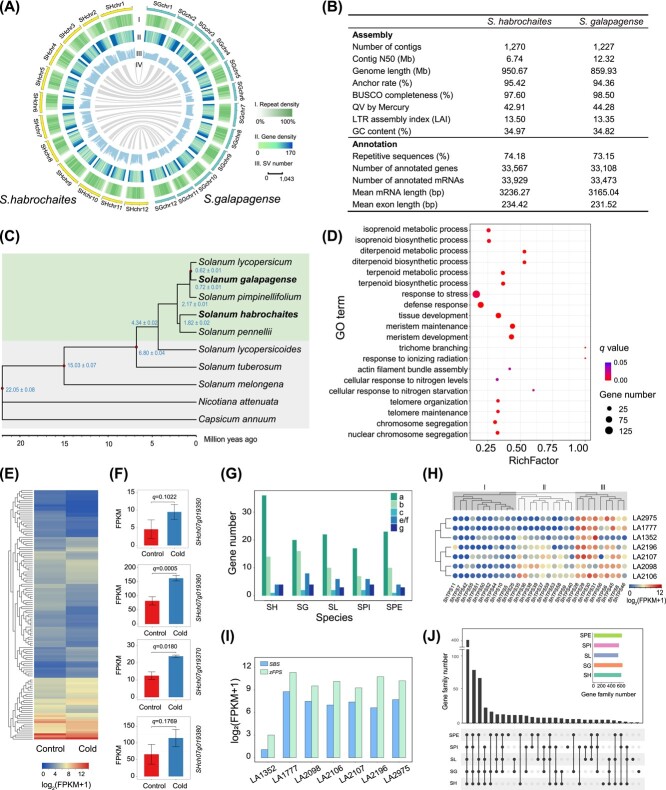
*Solanum habrochaites* and *Solanum galapagense* genomes. (A) Genomic landscape of *S. habrochaites* and *S. galapagense*. Densities of repeat sequences (I), genes (II), and SVs compared with *Solanum lycopersicum* (III) in 1-Mb windows, and syntenic blocks between *S. habrochaites* and *S. galapagense* (IV) are shown. (B) Statistics of the *S. habrochaites* and *S. galapagense* genome assemblies and annotations. (C) Phylogeny of 10 Solanaceae species with estimated divergence times. Red dots on the tree node indicate divergence times obtained from the TimeTree database (http://www.timetree.org/) that were used for calibration. (D) Gene Ontogeny (GO) terms enriched in genes overlapping with insertions and expansions in *S. habrochaites*. (E) Expression heat map of significantly up- or downregulated genes (*q* < .05) under cold stress in *S. habrochaites* with coding regions overlapping with insertions and expansions. (F) Expression of four tandemly duplicated *ShRCI3*s under cold stress. Error bars represent the standard deviation of three independent replicates. (G) Number of different TPS subfamily genes detected in five tomato species. (H, I) Expression of TPS genes (H) and *zFPS* (*SHch08g004680*) and *SBS* (*ShTPS45*, *SHch08g004730*) (I) in stem/petiole trichomes of seven different *S. habrochaites* accessions. (J) UpSet plot of RGA gene families among five tomato species. SH, *S. habrochaites*; SG, *S. galapagense*; SL, *S. lycopersicum*; SPI, *S. pimpinellifolium*; SPE, *S. pennellii*.

The *S. habrochaites* and *S. galapagense* genomes harbored 74.2% (705.2 Mb) and 73.2% (632.2 Mb) repetitive sequences, respectively, of which LTR retrotransposons accounted for 58.8 and 59.0% (Fig. 1B, Supplementary Data Table S1). A total of 33 567 and 33 108 protein-coding genes were predicted from the *S. habrochaites* and *S. galapagense* genome assemblies, respectively, and around 98% of the predicted genes could be annotated in public databases.

The phylogenetic tree constructed for *S. habrochaites*, *S. galapagense*, and eight other Solanaceae species using 3011 single-copy orthologous genes revealed that *S. habrochaites* was close to *Solanum pennellii* and that *S. galapagense* appeared close to *S. lycopersicum* (Fig. 1C), consistent with the previous phylogenomic study [4]. Molecular dating suggested that *S. habrochaites* diverged from *S. pennellii* around 1.82 million years ago (Mya), and *S. galapagense* and *S. lycopersicum* diverged around 0.62 Mya. A total of 16 666 gene families were shared among *S. habrochaites*, *S. galapagense*, *S. lycopersicum*, *S. pimpinellifolium*, and *S. pennellii*, and 366 and 190 were unique to *S. habrochaites* and *S. galapagense*, respectively (Supplementary Data Fig. S2). KEGG (Kyoto Encyclopedia of Genes and Genomes) pathway enrichment analysis suggested that these *S. habrochaites*-specific genes were significantly enriched with those involved in plant–pathogen interaction and in the MAPK signaling pathway, while the *S. galapagense*-specific genes were significantly enriched with those involved in zeatin and terpenoid biosynthesis.

To identify structure variants (SVs) relative to cultivated tomato, genome sequences of *S. habrochaites* and *S. galapagense* were compared with the *S. lycopersicum* genome (version SL4.0), according to the pipeline described in our previous study [5]. A total of 336 319 SVs with a total length of 257.9 Mb between *S. habrochaites* and *S. lycopersicum* and 98 443 SVs with a total length of 62.2 Mb between *S. galapagense* and *S. lycopersicum* were identified (Supplementary Data Fig. S3, Supplementary Data Table S2). The insertion and expansion regions in *S. habrochaites*, representing the *S. habrochaites*-specific sequences, overlapped with the coding regions of 5250 genes, which were significantly enriched with those involved in response to stress, defense response, terpenoid biosynthetic, and metabolic processes etc. (Fig. 1D). Coding regions of 1336 genes were found overlapping with the insertion and expansion regions of *S. galapagense*, and these genes were significantly enriched with those associated with defense response, pyrimidine nucleotide metabolism, and lipid metabolism etc. (Supplementary Data Fig. S4). These results suggested that the inserted and expanded genome regions in *S. habrochaites* and *S. galapagense* might contribute to the higher stress tolerance of the two wild tomato species. We found that the expression of 122 of these genes in *S. habrochaites* was significantly changed after cold treatment (Fig. 1E), including two of four tandem duplicates (*Shch07g019350*–*Shch07g019380*) homologous to *Arabidopsis rare cold-inducible protein 3* (*RIC3*) [6], which corresponded to only one copy (*Solyc07g049240*) in SL4.0 (Fig. 1F, Supplementary Data Fig. S5). The upregulation of *ShRIC3* genes by cold treatment suggested their potential roles in cold stress responses.

As mentioned above, the inserted/expanded genes in *S. habrochaites* were enriched in the terpenoid biosynthetic process. Terpenoids play roles in plant defense against pathogens and pests. Terpene synthases (TPSs) are key enzymes in generating terpenoids. A total of 59, 50, 43, 36, and 41 TPS genes were identified in genomes of *S. habrochaites*, *S. galapagense*, *S. lycopersicum*, *S. pimpinellifolium* (LA2093), and *S. pennellii* (LA0716), respectively (Fig. 1G, Supplementary Data Tables S3 and S4). Five TPS subfamilies, including TPS-a, -b, -c, -e/f and -g, were identified, and TPS-a was the most abundant (Supplementary Data Fig. S6). Since TPS-a members mainly encode sesquiterpene synthases, a remarkable expansion of this subfamily in *S. habrochaites* suggested potentially diverse or unique sesquiterpene synthesis in this species. Eighteen *ShTPS*s were not expressed in any of the investigated tissues, including leaf, stem, root, flower, and fruit, while the remainder were mainly expressed in a tissue-specific manner (Supplementary Data Fig. S7). Trichomes play roles in plant defense by providing specialized metabolites, including terpenes. Nearly half of the *ShTPS*s were expressed in stem/petiole trichomes of seven *S. habrochaites* accessions, and these *ShTPS*s were further divided into three groups based on their expression patterns (Fig. 1H). The various TPS expression patterns probably contributed to the diversity of terpene composition in these accessions [7]. A novel sesquiterpene biosynthesis pathway involving SBS (santalene and bergamotene synthase, a TPS-e/f member) and zFPS (*Z*-isoprenyl pyrophosphate synthase) has been proposed in *S. habrochaites* [8]. Our results showed that *SBS* and *zFPS* had similar expression levels in the seven *S. habrochaites* accessions, except LA1352, suggesting both conserved and diverged sesquiterpene biosynthesis in these accessions (Fig. 1I).

The wild relatives of tomato are the main gene source for tomato resistance breeding [9]. To explore the reservoir of resistance genes in tomato species, resistance gene analogs (RGAs) were identified in genomes of *S. habrochaites*, *S. galapagense*, *S. lycopersicum*, *S. pimpinellifolium*, and *S. pennellii*. In total, 4668 RGAs were detected in these five species, including 2482 receptor-like protein kinases (RLKs), 831 nucleotide binding site (NBS)-encoding proteins and 391 receptor-like proteins (RLPs) (Supplementary Data Table S5). Gene family analysis indicated that 401 gene families (2685 genes) were shared in all five tomato species, while 187 gene families (919 genes) were not found in *S. lycopersicum* (Fig. 1J). In addition, 163 and 36 RGAs were found in the insertion/expansion regions of *S. habrochaites* and *S. galapagense*, respectively. These extra RGAs might contribute to the high disease resistance of the two species.

In summary, the high-quality genome assemblies of *S. habrochaites* and *S. galapagense* provide robust references, in particular, new gene sources of stress tolerance and terpene biosynthesis for functional genomic research and genetic improvement in tomato.

## Acknowledgements

This work was supported by grants from the National Natural Science Foundation of China (32170395), the US National Science Foundation (IOS-1855585), the Foundations of Hubei Hongshan Laboratory (2021hszd017), and the Key Laboratory of Plant Germplasm Enhancement and Specialty Agriculture, Wuhan Botanical Garden, Chinese Academy of Sciences.

## Author contributions

L.G., Z.F., X.Y., and S.G conceived the project. Y.S., M.Q., S.G., and C.H. performed the experiments. X.Y. and M.Q. analyzed the data and wrote the manuscript. L.G., Z.F., and S.G revised the manuscript.

## Data availability

Raw sequencing data and genome assemblies have been deposited in the Genome Sequence Archive (GSA) under the BioProject accession number PRJCA008297.

## Conflict of interest

The authors declare no conflicts of interest.

## Supplementary data

Supplementary data is available at *Horticulture Research* online.

## Supplementary Material

Web_Material_uhac139
