# Supplementary material for: Chromosome-scale genome assemblies of wild tomato relatives Solanum habrochaites and Solanum galapagense reveal structural variants associated with stress tolerance and terpene biosynthesis
Source: Hortic Res. 2022 Jun 20;9:uhac139. doi: 10.1093/hr/uhac139 (PMC9350829; doi:10.1093/hr/uhac139)
Supplement: Web_Material_uhac139 [file web_material_uhac139.docx]

**Chromosome-scale genome assemblies of wild tomato relatives *Solanum habrochaites* and *S. galapagense* reveal structural variants associated with stress tolerance and terpene biosynthesis**

Xiaofen Yu^1,2,†^, Minghao Qu^1,3,†^, Yanna Shi^4^, Chenlu Hao^1,3^, Sumin Guo^1,*^,
Zhangjun Fei^5,6,*^ and Lei Gao^1,2,*^

^1^ CAS Key Laboratory of Plant Germplasm Enhancement and Specialty Agriculture, Wuhan Botanical Garden, Innovative Academy of Seed Design, Chinese Academy of Sciences, Wuhan, China

^2^ Hubei Hongshan Laboratory, Wuhan, China

^3^ University of Chinese Academy of Sciences, Beijing, China

^4^ Zhejiang Provincial Key Laboratory of Horticultural Plant Integrative Biology, Zhejiang University, Hangzhou, China

^5^ Boyce Thompson Institute, Cornell University, Ithaca, NY, USA

^6^ U.S. Department of Agriculture-Agricultural Research Service, Robert W. Holley Center for Agriculture and Health, Ithaca, NY, USA

*Correspondence (Tel/fax +86-27-8770-0860; emails: guosumin@wbgcas.cn [SG], zf25@cornell.edu [ZF] and leigao@wbgcas.cn [LG])

^†^ These authors contributed equally to this work.

**Supplementary material**

[Supplementary note](#_Toc103350027)

[Plant materials and sampling 3](#_Toc103350028)

[Library construction and sequencing 3](#_Toc103350029)

[Genome assembly and quality assessment 3](#_Toc103350030)

[Genome annotation 4](#_Toc103350031)

[Phylogenomic analysis and estimation of divergence times 4](#_Toc103350032)

[Structure variant detection 5](#_Toc103350033)

[Gene expression analysis of *S. habrochaites* under cold stress 5](#_Toc103350034)

[Terpene synthase gene identification and expression analysis 5](#_Toc103350035)

[Resistance gene analog identification 6](#_Toc103350036)

[References 7](#_Toc103350037)

[**Supplementary figures** 9](#_Toc103350038)

[Figure S1 9](#_Toc103350039)

[Figure S2 10](#_Toc103350040)

[Figure S3 11](#_Toc103350041)

[Figure S4 12](#_Toc103350042)

[Figure S5 13](#_Toc103350043)

[Figure S6 14](#_Toc103350044)

[Figure S7 15](#_Toc103350045)

[**Supplementary tables** 16](#_Toc103350046)

[Table S1 16](#_Toc103350047)

[Table S2 17](#_Toc103350048)

[Table S3. 18](#_Toc103350049)

[Table S4 20](#_Toc103350050)

[Table S5 22](#_Toc103350051)

# Supplementary note

## Plant materials and sampling

Seeds of *Solanum habrochaites* (LA0407) and *S. galapagense* (LA0317) were obtained from the Tomato Genetics Resource Center (TGRC), and plants were grown in the greenhouse at Wuhan Botanical Garden, Chinese Academy of Sciences (Wuhan, Hubei Province), at 21-24°C with a 11 h light/13 h dark cycle.

For genome sequencing, fresh leaves were collected, immediately frozen in liquid nitrogen and stored at -80°C. For RNA sequencing, leaves, stems, roots, flowers and fruits were collected at the reproductive growth stage, immediately frozen in liquid nitrogen and stored at -80°C.

## Library construction and sequencing

PacBio SMRT libraries were constructed following the standard SMRTbell library preparation protocol and sequenced on a PacBio Sequel II platform (Pacific Biosciences, Menlo Park, USA). High-fidelity (HiFi) reads were generated using the ccs software (https://github.com/PacificBiosciences/ccs) with parameters ‘min-passes=3, min-rq=0.99’. A total of 27.8 Gb and 23.8 Gb HiFi sequences with an average length of 17.9 and 16.9 kb were generated for *S. habrochaites* and *S. galapagense*, respectively.

Illumina paired-end libraries with insert sizes of ~350 bp were constructed using the TruSeq Nano DNA HT Sample Prep Kit (Illumina, USA) following the manufacturer’s instructions. The libraries were then sequenced on an Illumina HiSeq X Ten system to generate 150-bp paired-end reads. Quality control of the raw sequencing data was performed using fastp v0.22.0 [1].

Hi-C libraries were constructed according to the standard protocol and sequenced on Illumina HiSeq X Ten system with the paired-end mode and read length of 150 bp. A total of 151.6 Gb and 107.3 Gb reads were generated, corresponding to 159.5× and 124.9× of *S. habrochaites* and *S. galapagense* genomes, respectively.

Total RNA was extracted from leaves, stems, roots, flowers and fruits using Tiangen RNAprep pure plant kit (Tiangen Biotech, China). RNA-seq libraries were constructed using NEBNext UltraTM RNA Library Prep Kit (NEB, USA) according to manufacturer’s instructions and sequenced on an Illumina Novaseq 6000 platform (Illumina, USA) to generate 150-bp paired-end reads.

## Genome assembly and quality assessment

Genome sizes of *S. habrochaites* LA0407 and *S. galapagense* LA0317 were estimated using a k-mer based approach with Jellyfish v2.2.10 [2] and GCE v1.0.2 [3], which were 963 Mb for *S. habrochaites* and 876 Mb for *S. galapagense*. HiFi reads were *de novo* assembled into contigs using hifiasm v0.15.3 [4]. The potential contaminations in resulted contigs were screened by searching against the GenBank nucleotide database using BLASTN. Hi-C sequencing data were filtered using HICUP v0.8.0 [5], and the cleaned data were aligned to the assembled contigs using Juicer v1.9.9 [6]. The resulting alignments were used to construct pseudochromosomes using 3D-DNA v180114 [7], which were further sorted and corrected using Juicebox v1.11.08 [8].

Several methods were used to extensively evaluate the quality of the two genome assemblies. The genome assemblies was evaluated using the LTR Assembly Index (LAI) [9] and BUSCO v5.0 with the ‘embryophyta_odb10’ database [10]. Approximately 99.8% of Illumina short reads could be mapped back to the genome assemblies using BWA v0.7.17 [11]. Additionally, Merqury v2020-01-29 [12] was used to assess the consensus quality values (QV) of the genome assemblies.

## Genome annotation

Repeat libraries for the two genome assemblies were constructed using RepeatModeler v2.0.1 [13] integrated with LTRharvest [14] and LTR_retriever [15]. LTR_FINDER v1.07 [16] was used to predict the location and structure of full-length LTR retrotransposons. The repeat libraries were utilized to scan the genome assemblies with RepeatMasker v4.1.1 (http://www.repeatmasker.org) to identify repetitive sequences in each genome assembly.

A combination of *ab initio*, homologue-based, and RNA-seq-based predictions were adopted to predict protein-coding genes in the two genome assemblies. For RNA-seq-based predictions, RNA-seq reads were aligned and assembled using HISAT2 v2.2.1 [17] and StringTie2 v2.1.6 [18]. TransDecoder v5.5.0 (http://transdecoder.github.io) was used to predict coding regions from the assembled transcripts. AUGUSTUS v3.3.3 [19] was used for *ab initio* predictions. For homologue-based predictions, protein sequences of five species (*Arabidopsis thaliana*, *S. lycopersicum*, *S. tuberosum*, *Nicotiana attenuata* and *Capsicum annuum*) were mapped to each of the two genome assemblies using TBLASTN [20] with a cutoff e-value of 1e-5, and homologous genes were identified using exonerate v2.2.0 [21]. Finally, all the predictions were integrated using MAKER v3.01.04 [22] with default parameters to produce a consensus set of gene models for each genome. Genes without a ‘canonical’ ATG start codon or the stop codons TAA, TAG or TGA were excluded.

The predicted protein-coding genes were functionally annotated based on five public databases, including GO (http://geneontology.org/), KEGG (https://www.kegg.jp/), GenBank nr (https://www.ncbi.nlm.nih.gov/), Uniprot (https://www.uniprot.org/) and Interpro (http://www.ebi.ac.uk/interpro/). Alignments were performed using DIAMOND v2.0.13.151 [23]. Over 97% of the predicted genes in *S. habrochaites* and *S. galapagense* could be annotated in these databases.

## Phylogenomic analysis and estimation of divergence times

OrthoFinder v2.5.2 [24] was used to infer a matrix of orthologous groups (gene families) among the selected genomes. Multiple sequence alignments were performed using MUSCLE v3.8.31 [25] for single-copy orthologous genes. A maximum likelihood phylogenetic tree was constructed using single-copy orthologous genes with RAxML v8.2.12 [26]. Divergence times between species were estimated using the MCMC tree program implemented in PAML v4.9i [27]. KEGG pathways significantly enriched in the *S. habrochaites*- or *S. galapagense*-specific genes were identified using Fisher's exact test with an adjusted *p* value (*q* value) < 0.05.

## Structure variant detection

To identify SVs, chromosome sequences of *S. habrochaites* or *S. galapagense* were aligned to the Heinz 1706 genome (SL4.0) using Minimap2 [28] with the parameter ‘-ax asm10’ for *S. habrochaites* or ‘-ax asm5’ for *S. galapagense*. The unique alignments were extracted and used for SV calling using Assemblytics [29]. The identified SVs covering or adjacent to gaps regions in either of the two compared genomes were filtered out. Furthermore, for each of the two comparisons (*S. habrochaites* vs. Heinz 1706 and *S. galapagense* vs. Heinz 1706), Illumina reads from the two analyzed genomes were mapped to both genomes, respectively, using BWA v0.7.17 [11]. The resulting alignments were used to validate or refine breakpoints of the identified SVs using our in-house scripts of the published pipeline (https://github.com/GaoLei-bio/SV) [30]. Genes with coding sequences overlapping with SV regions were extracted using BEDTools v2.29.1 [31].

## Gene expression analysis of *S. habrochaites* under cold stress

To analyze gene expression patterns under cold stress in *S. habrochaites*, raw RNA-seq reads were downloaded from NCBI Sequence Read Archive (SRA) (accession number ERP016476), and processed to remove adapter and low-quality sequences using fastp v0.22.0 [1]. The resulting cleaned reads were mapped to the assembled *S. habrochaites* genomes using STAR v2.7.9a [32] with parameters: --outFilterMultimapNmax 1 --outFilterType BySJout --outSAMattrIHstart 0 --outSAMstrandField intronMotif --alignIntronMin 20 --alignIntronMax 1000 --outFilterMismatchNmax 2 --outSJfilterReads Unique --limitSjdbInsertNsj 500 --alignMatesGapMax 500. Read counting was performed using featureCounts v2.0.2 [33]. Genes differentially expressed between the control and cold stressed samples were identified using DESeq2 [34] with an adjusted *p* value (*q* value) cutoff of 0.05. FPKM (Fragments per kilo base of transcript per million mapped fragments) were calculated by StringTie v2.1.6 [18].

## Terpene synthase gene identification and expression analysis

Both HMM- and BLAST-based methods were used to identify terpene synthases (TPSs) in *S. habrochaites*, *S. galapagense*, *S. lycopersicum*, *S. pimpinellifolium* and *S. pennellii*. Two Pfam domains, PF01397 and PF03936, were used to search each proteome with HMMER v3.2 (http://eddylab.org/software/hmmer/hmmer.org) with an e-value cutoff of 1e-20. Known functional TPSs in *S. lycopersicum* (SL3.0) were also used as queries for a BLASTP search with e-value cutoff of 1e-5. All the identified TPS candidates were further validated through phylogenetic analysis.

To analyze *TPS* gene expression patterns in trichomes, raw RNA-seq reads of stem/petiole trichomes from seven *S. habrochaites* accessions were downloaded from NCBI SRA (accession number SRP013118). Adapter and low-quality sequences of raw reads were removed to obtain clean reads by Fastp v0.22.0 [1], and the clean reads were mapped to the assembled *S. habrochaites* genome using HISAT2 v2.2.1 [17] with default parameters. Gene expression levels were then calculated using StringTie v2.1.6 [18].

## Resistance gene analog identification

Genome-wide resistance gene analogs (RGAs) were identified using the RGAugury pipeline [35]. The identified RGA candidates were classified into four major families: NBS-encoding proteins, receptor-like protein kinases (RLKs), receptor-like proteins (RLPs) and proteins with both transmembrane domain and coiled-coil domain (TM-CCs). NBS-encoding proteins were further divided into several subgroups according to their domain architecture, namely NBS (with only an NB-ARC domain), CNL (with coils, NB-ARC and leucine-rich repeat domains), CN (with coils and NB-ARC domains), TNL (with TIR, NB-ARC and leucine-rich repeat domains), TN (with TIR and NB-ARC domains), NL (with NB-ARC and leucine-rich repeat domains), TX (with TIR and unclassified domains), and Other.

## References

1. Chen S, Zhou Y, Chen Y, Gu J. fastp: an ultra-fast all-in-one FASTQ preprocessor. *Bioinformatics*. 2018;**34**:i884-90.

2. Marçais G, Kingsford C. A fast, lock-free approach for efficient parallel counting of occurrences of k-mers. *Bioinformatics*. 2011;**27**:764-70.

3. Liu B, Shi Y, Yuan J *et al.* Estimation of genomic characteristics by analyzing k-mer frequency in *de novo* genome projects. *arXiv*. 2013;1308.2012 [q-bio]

4. Cheng H, Concepcion GT, Feng X *et al*. Haplotype-resolved *de novo* assembly using phased assembly graphs with hifiasm. *Nat Methods*. 2021;**18**:170-5.

5. Wingett S, Ewels P, Furlan-Magaril M *et al.* HiCUP: pipeline for mapping and processing Hi-C data. *F1000Res*. 2015;**4**:1310.

6. Durand NC, Shamim MS, Machol I *et al.* Juicer provides a one-click system for analyzing loop-resolution Hi-C experiments. *Cell Syst*. 2016;**3**:95-8.

7. Dudchenko O, Batra Sanjit S, Omer Arina D *et al.* *De novo* assembly of the *Aedes aegypti* genome using Hi-C yields chromosome-length scaffolds. *Science*. 2017;**356**:92-5.

8. Robinson JT, Turner D, Durand NC *et al*. Juicebox.js provides a cloud-based visualization system for Hi-C data. *Cell Syst*. 2018;**6**:256-8.

9. Ou S, Chen J, Jiang N. Assessing genome assembly quality using the LTR Assembly Index (LAI). *Nucleic Acids Res*. 2018;**46**:e126.

10. Manni M, Berkeley MR, Seppey M *et al*. BUSCO update: Novel and streamlined workflows along with broader and deeper phylogenetic coverage for scoring of eukaryotic, prokaryotic, and viral genomes. *Mol Biol Evol*. 2021;**38**:4647-54.

11. Li H, Durbin R. Fast and accurate short read alignment with Burrows-Wheeler transform. *Bioinformatics*. 2009;**25**:1754-60.

12. Rhie A, Walenz B, Koren S, Phillippy A. Merqury: Reference-free quality, completeness, and phasing assessment for genome assemblies. *Genome Biol*. 2020;**21**:245.

13. Flynn JM, Hubley R, Goubert C *et al.* RepeatModeler2 for automated genomic discovery of transposable element families. *Proc Natl Acad Sci U S A*. 2020;**117**:9451-57.

14. Ellinghaus D, Kurtz S, Willhoeft U. LTRharvest, an efficient and flexible software for *de novo* detection of LTR retrotransposons. *BMC Bioinformatics*. 2008;**9**:18.

15. Ou S, Jiang N. LTR_retriever: A highly accurate and sensitive program for identification of long terminal repeat retrotransposons. *Plant Physiol*. 2018;**176**:1410-22.

16. Xu Z, Wang H. LTR_FINDER: an efficient tool for the prediction of full-length LTR retrotransposons. *Nucleic Acids Res*. 2007;**35**:W265-8.

17. Kim D, Paggi JM, Park C *et al*. Graph-based genome alignment and genotyping with HISAT2 and HISAT-genotype. *Nat Biotechnol*. 2019;**37**:907-15.

18. Kovaka S, Zimin AV, Pertea GM *et al*. Transcriptome assembly from long-read RNA-seq alignments with StringTie2. *Genome Biol*. 2019; **20:** 278.

19. Stanke M, Diekhans M, Baertsch R, Haussler D. Using native and syntenically mapped cDNA alignments to improve *de novo* gene finding. *Bioinformatics*. 2008;**24**:637-44.

20. Altschul S, Gish W, Miller W *et al*. Basic local aligment search tool. *J Mol Biol*. 1990;**215**:403-10.

21. Slater GSC, Birney E. Automated generation of heuristics for biological sequence comparison. *BMC Bioinformatics*. 2005;**6**:31.

22. Cantarel BL, Korf I, Robb SMC *et al.* MAKER: an easy-to-use annotation pipeline designed for emerging model organism genomes. *Genome Res*. 2008;**18**:188-96.

23. Buchfink B, Reuter K, Drost HG. Sensitive protein alignments at tree-of-life scale using DIAMOND. *Nat Methods*. 2021;**18**:366-68.

24. Emms DM, Kelly S. OrthoFinder: phylogenetic orthology inference for comparative genomics. *Genome Biol*. 2019;**20**:238.

25. Edgar RC. MUSCLE: multiple sequence alignment with high accuracy and high throughput. *Nucleic Acids Res*. 2004;**32**:1792-97.

26. Stamatakis A. RAxML version 8: a tool for phylogenetic analysis and post-analysis of large phylogenies. *Bioinformatics*. 2014;**30**:1312-13.

27. Yang Z. PAML 4: Phylogenetic analysis by maximum likelihood. *Mol Biol Evol*. 2007;**24**:1586-91.

28. Li H. Minimap2: pairwise alignment for nucleotide sequences. *Bioinformatics*. 2018;**34**:3094-100.

29. Nattestad M, Schatz MC. Assemblytics: a web analytics tool for the detection of variants from an assembly. *Bioinformatics*. 2016;**32**:3021-23.

30. Wang X, Gao L, Jiao C *et al.* Genome of *Solanum pimpinellifolium* provides insights into structural variants during tomato breeding. *Nat Commun*. 2020;**11**:5817.

31. Quinlan AR, Hall IM. BEDTools: a flexible suite of utilities for comparing genomic features. *Bioinformatics*. 2010;**26**:841-42.

32. Dobin A, Gingeras TR. Mapping RNA-seq reads with STAR. *Curr Protoc Bioinformatics*. 2015;51:11.14.1-19.

33. Liao Y, Smyth GK, Shi W. featureCounts: an efficient general purpose program for assigning sequence reads to genomic features. *Bioinformatics*. 2014;**30**:923-30.

34. Love MI, Huber W, Anders S. Moderated estimation of fold change and dispersion for RNA-seq data with DESeq2. *Genome Biol*. 2014;**15**:550.

35. Li P, Quan X, Jia G *et al*. RGAugury: a pipeline for genome-wide prediction of resistance gene analogs (RGAs) in plants. *BMC Genomics*. 2016;**17**:852.

# Supplementary figures


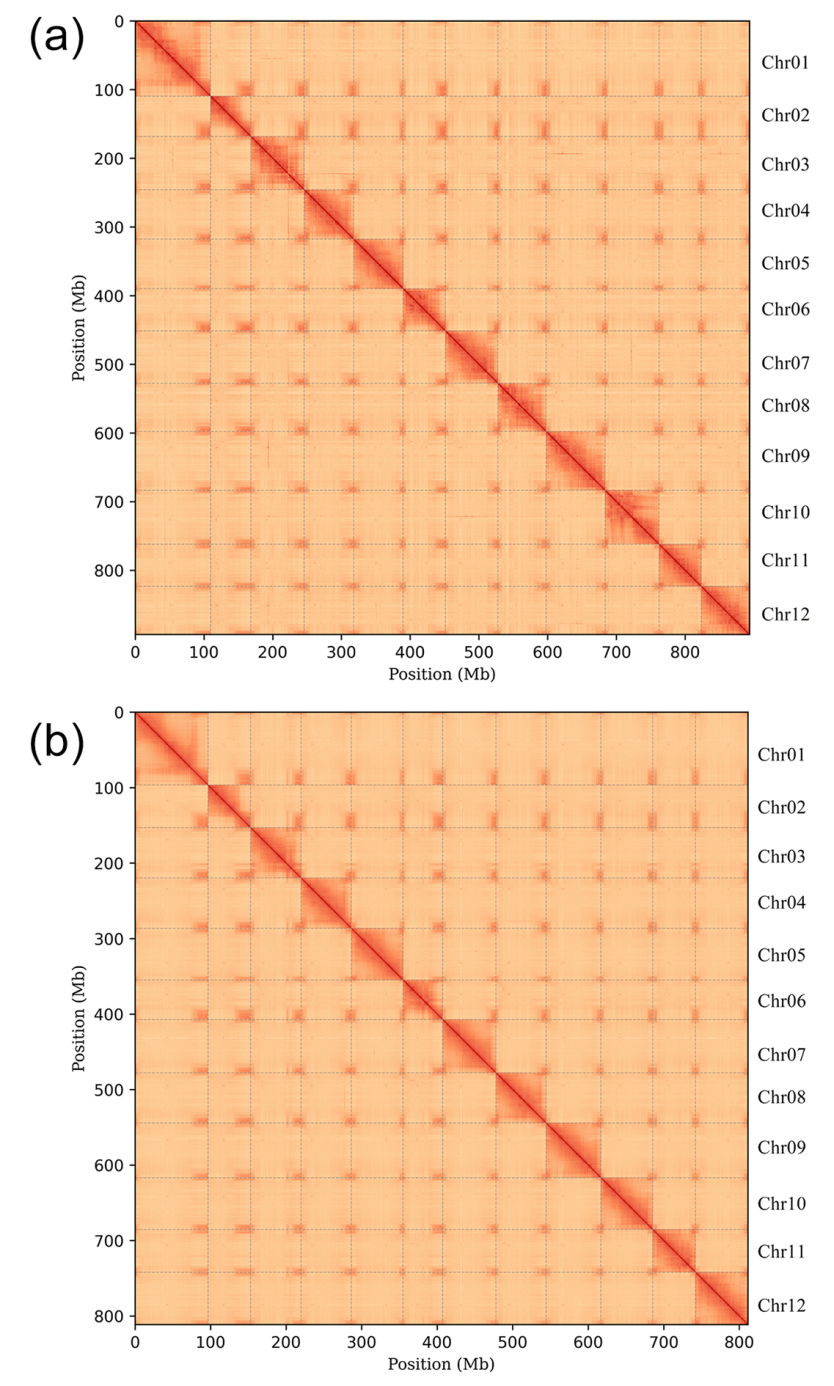


Figure S1**. Hi-C interactions among the 12 chromosomes of *S. habrochaites* (a) and *S. galapagense* (b)**. Strong interactions are indicated in dark red while weak interactions are indicated in yellow.


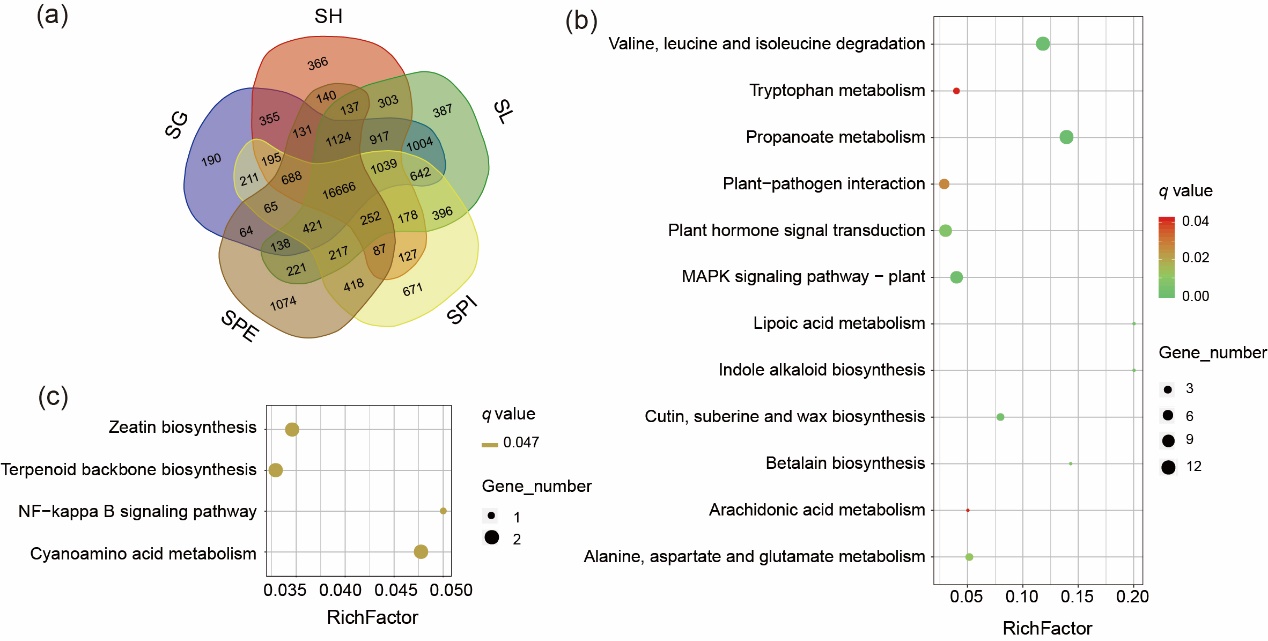


Figure S2**. Gene family and KEGG enrichment analysis.** (a) Venn diagram of gene families of five tomato species. SH, *S. habrochaites*; SG, *S. galapagense*; SL, S. *lycopersicum*; SPI, *S. pimpinellifolium*; SPE, *S. pennellii*. (b) KEGG pathways enriched in unique genes of *S. habrochaites*. (c) KEGG pathways enriched in unique genes of *S. galapagense*. A total of 366 gene families (734 genes) in *S. habrochaites* and 190 unique gene families (308 genes) in *S. galapagense* were analyzed.


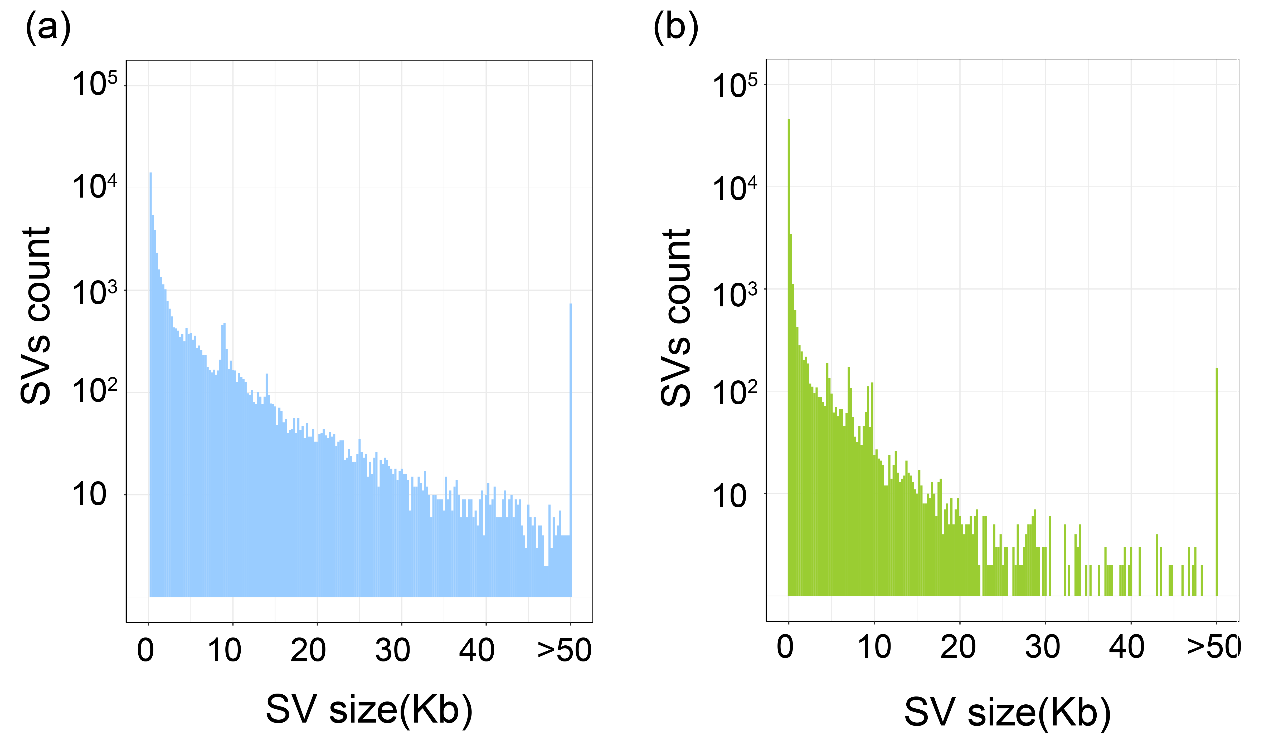


Figure S3**. SV sizes and numbers of *S. habrochaites* (a) and *S. galapagense* (b).**


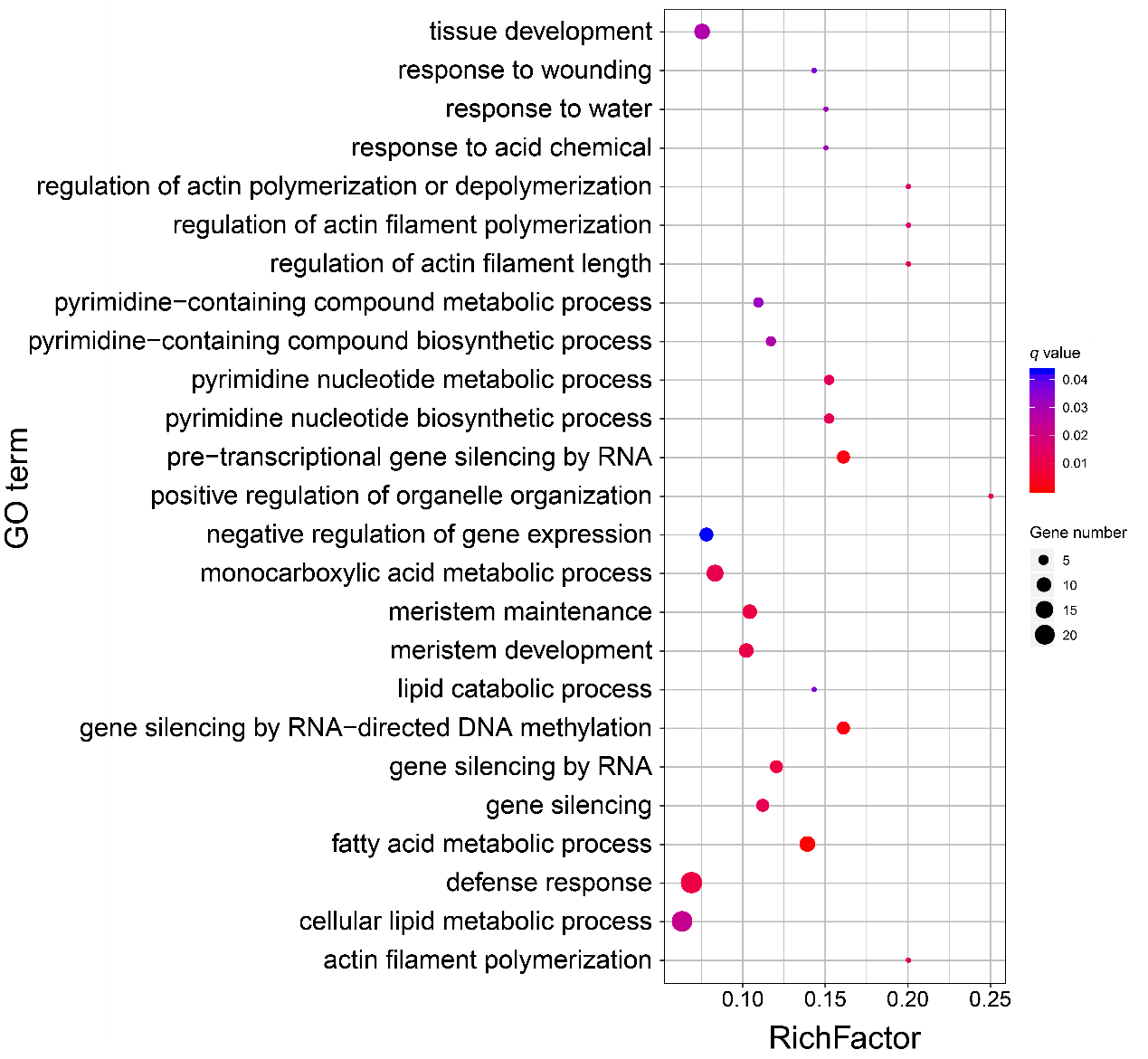


Figure S4**.** **Functional enrichment analysis of genes overlapping with insertions and expansions in *S. galapagense*.** Biological processes with *q* values less than 0.05 are shown.

**
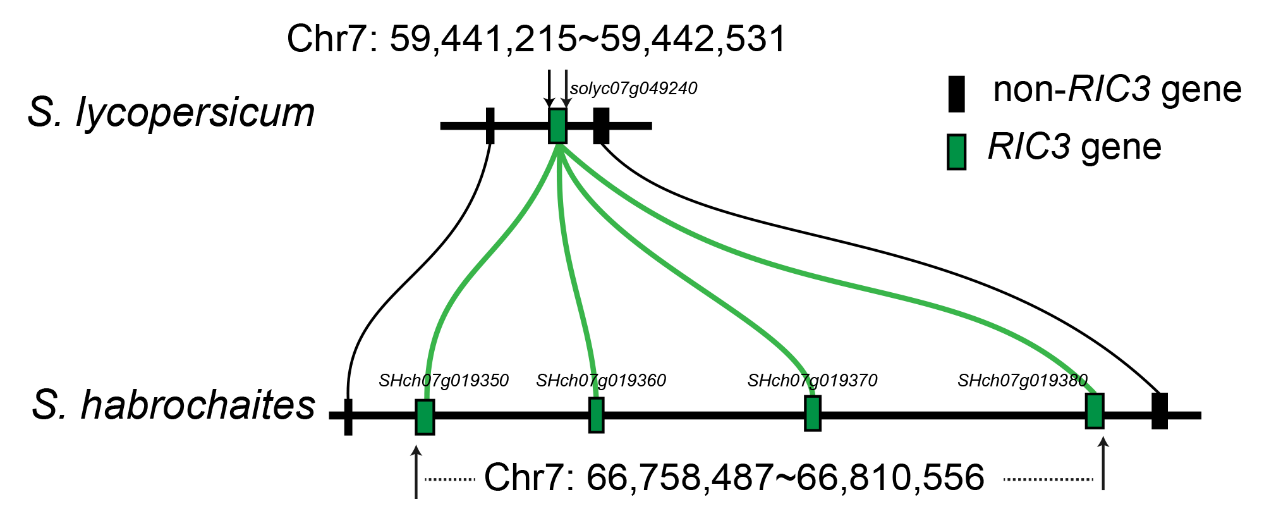
**

Figure S5**.** **Copy number variant of *RIC3* genes in *S. lycopersicum* and *S. habrochaites*.**

**
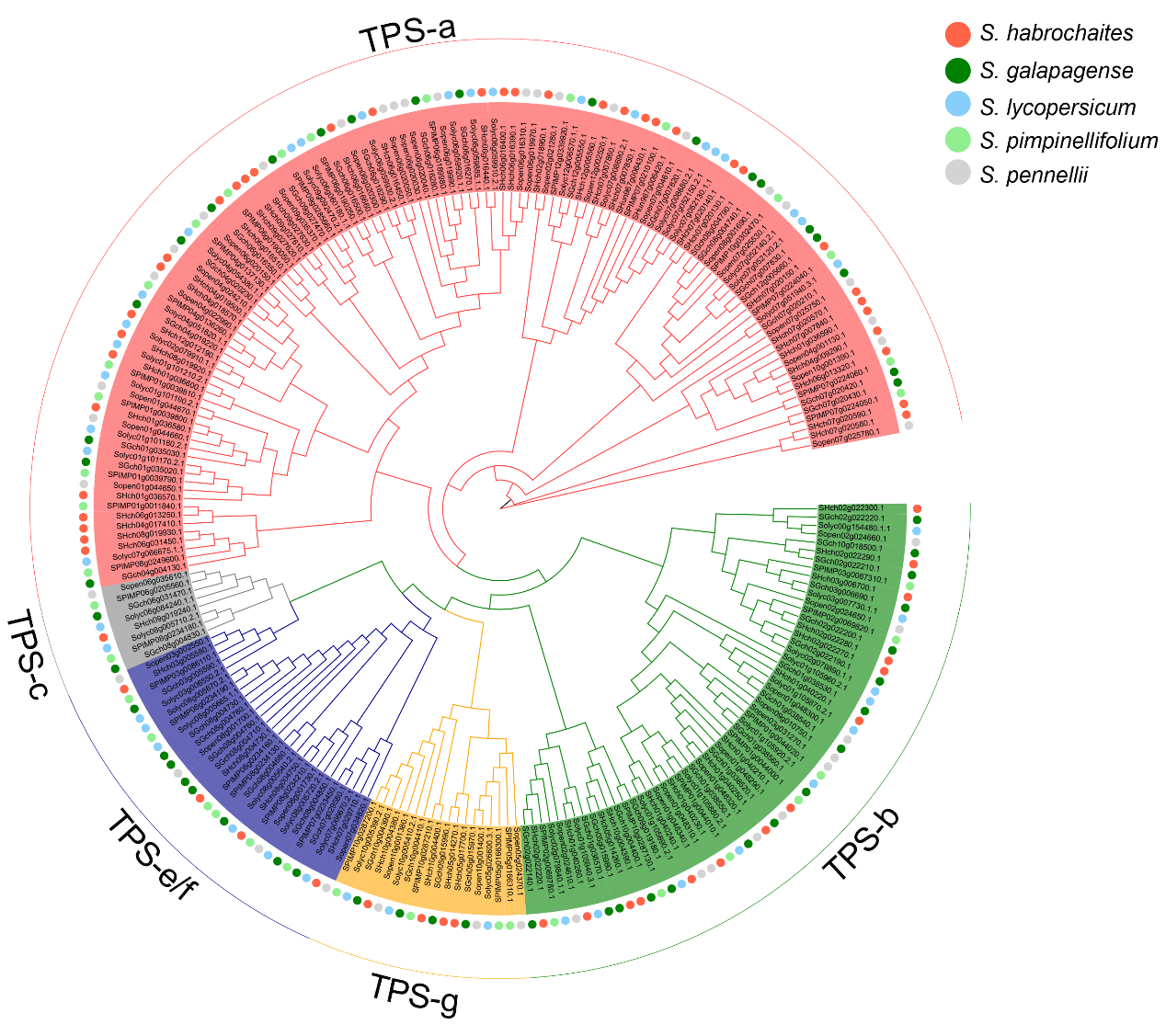
**

Figure S6**.** **Phylogenetic tree of TPS genes from *S. habrochaites*, *S. galapagense*, *S. lycopersicum*, *S. pimpinellifolium* and *S. pennelli*.**


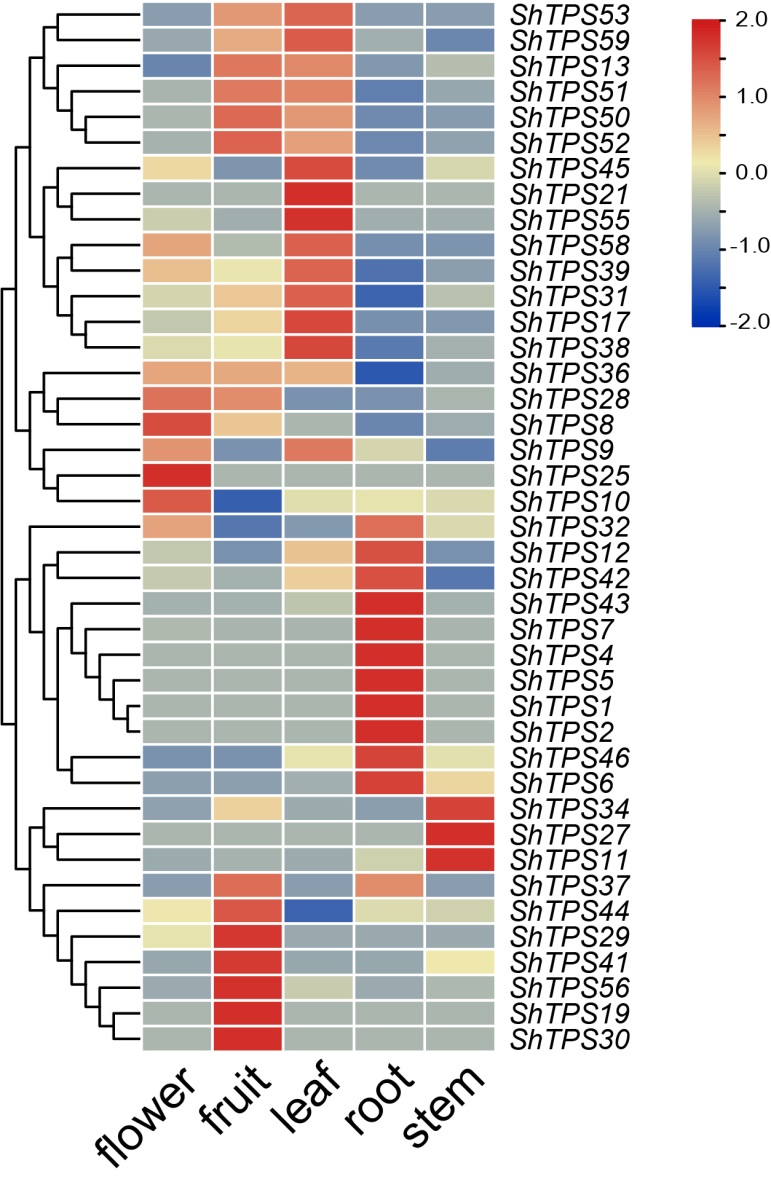


Figure S7**.** **Expression profiles of TPS genes in five tissues of *S. habrochaites*.** Row scaling was applied. TPS genes not expressed in any of the five tissues are not shown. Red and blue colors indicate relatively high and low expression levels, respectively.

# Supplementary tables

Table S1**. Summary statistics of repeat sequences in the *S. habrochaites* and *S. galapagense* genomes.**

| **Type** | ***S. habrochaites*** | | ***S. galapagense*** | |
| --- | --- | --- | --- | --- |
|  | Length (bp) | Percentage | Length (bp) | Percentage |
| LTR elements | 414,364,163 | 43.59% | 373,125,140 | 43.39% |
| Ty1/Copia | 71,228,937 | 7.49% | 66,585,067 | 7.74% |
| Gypsy/DIRS1 | 264,572,076 | 27.83% | 235,256,409 | 27.36% |
| LINEs | 12,416,690 | 1.31% | 10,400,035 | 1.21% |
| DNA transposons | 8,449,116 | 0.89% | 7,284,624 | 0.85% |
| Simple repeats | 9,937,788 | 1.05% | 7,152,585 | 0.83% |
| **Total** | 705,184,477 | 74.18% | 632,159,384 | 73.51% |

Table S2**. Summary of detected SVs in the genomes of *S. habrochaites* and *S. galapagense* compared with that of *S. lycopersicum*.**

| **SV type** | ***S. habrochaites*** | ***S. galapagense*** |
| --- | --- | --- |
| Deletion | 147,429 | 42,285 |
| Insertion | 162,823 | 52,327 |
| Substitution | 2,008 | 509 |
| Repeat expansion | 12,542 | 1,706 |
| Repeat contraction | 11,206 | 1,513 |
| Tandem expansion | 138 | 52 |
| Tandem contraction | 173 | 51 |
| **Total** | 336,319 | 98,443 |

Table S3. **TPS genes identified in genomes of five tomato species**

| **Subfamily** | | ***S. habrochaites*** | ***S. galapagense*** | ***S. lycopersicum*** | ***S. pimpinellifolium*** | ***S. pennellii*** |
| --- | --- | --- | --- | --- | --- | --- |
| **TPS-a** | SHch01g036570 | | SGch01g035020 | Solyc01g101170 | SPIMP01g0011840 | Sopen01g044650 |
|  | SHch01g036580 | | SGch01g035030 | Solyc01g101180 | SPIMP01g0039790 | Sopen01g044660 |
|  | SHch01g036590 | | SGch04g004130 | Solyc01g101190 | SPIMP01g0039800 | Sopen01g044670 |
|  | SHch01g036600 | | SGch04g019220 | Solyc01g101210 | SPIMP01g0039810 | Sopen02g021280 |
|  | SHch02g018950 | | SGch04g020230 | Solyc02g079910 | SPIMP04g0136260 | Sopen04g001130 |
|  | SHch04g009290 | | SGch06g016270 | Solyc04g051620 | SPIMP04g0137130 | Sopen04g022990 |
|  | SHch04g017410 | | SGch06g016280 | Solyc04g054380 | SPIMP06g0189980 | Sopen04g024210 |
|  | SHch04g018570 | | SGch06g016290 | Solyc06g059885 | SPIMP06g0190050 | Sopen06g016310 |
|  | SHch04g019500 | | SGch06g016350 | Solyc06g059910 | SPIMP06g0190250 | Sopen06g019970 |
|  | SHch06g013250 | | SGch06g016500 | Solyc06g059920 | SPIMP07g0210100 | Sopen06g019990 |
|  | SHch06g013320 | | SGch07g007820 | Solyc06g059930 | SPIMP07g0224040 | Sopen06g020030 |
|  | SHch06g016390 | | SGch07g007830 | Solyc06g060180 | SPIMP07g0224050 | Sopen06g020040 |
|  | SHch06g016400 | | SGch07g020210 | Solyc07g008680 | SPIMP07g0224060 | Sopen06g020050 |
|  | SHch06g016440 | | SGch07g020420 | Solyc07g008690 | SPIMP08g0249600 | Sopen06g020150 |
|  | SHch06g016450 | | SGch07g020430 | Solyc07g052130 | SPIMP09g0285660 | Sopen06g020300 |
|  | SHch06g016510 | | SGch08g004740 | Solyc07g052140 | SPIMP10g0302470 | Sopen07g004610 |
|  | SHch06g016660 | | SGch08g004790 | Solyc07g051940 | SPIMP12g0339920 | Sopen07g025530 |
|  | SHch06g031450 | | SGch09g027470 | Solyc07g052120 |  | Sopen07g025750 |
|  | SHch07g007840 | | SGch12g005550 | Solyc07g052150 |  | Sopen07g025780 |
|  | SHch07g007850 | | SGch12g005660 | Solyc07g066675 |  | Sopen08g001690 |
|  | SHch07g007860 | |  | Solyc09g092470 |  | Sopen09g035370 |
|  | SHch07g020130 | |  | Solyc12g006570 |  | Sopen10g001390 |
|  | SHch07g020140 | |  |  |  | Sopen12g002520 |
|  | SHch07g020150 | |  |  |  |  |
|  | SHch07g020570 | |  |  |  |  |
|  | SHch07g020580 | |  |  |  |  |
|  | SHch07g020590 | |  |  |  |  |
|  | SHch08g019920 | |  |  |  |  |
|  | SHch08g019930 | |  |  |  |  |
|  | SHch09g027610 | |  |  |  |  |
|  | SHch09g027620 | |  |  |  |  |
|  | SHch09g027630 | |  |  |  |  |
|  | SHch12g005560 | |  |  |  |  |
|  | SHch12g012190 | |  |  |  |  |
|  | SHun967g006420 | |  |  |  |  |
|  | SHun967g006430 | |  |  |  |  |
| **TPS-b** | SHch01g040210 | | SGch01g038520 | Solyc01g105870 | SPIMP01g0044000 | Sopen01g048290 |
|  | SHch01g040220 | | SGch01g038530 | Solyc01g105880 | SPIMP01g0044010 | Sopen01g048300 |
|  | SHch01g040230 | | SGch01g038540 | Solyc01g105890 | SPIMP01g0044020 | Sopen01g048310 |
|  | SHch01g040240 | | SGch01g038550 | Solyc01g105920 | SPIMP02g0069780 | Sopen01g048320 |
|  | SHch01g040260 | | SGch01g038560 | Solyc01g105940 | SPIMP02g0069820 | Sopen01g048340 |
|  | SHch01g040250 | | SGch01g038570 | Solyc01g105960 | SPIMP03g0087310 | Sopen02g024610 |
|  | SHch02g022220 | | SGch02g022140 | Solyc02g079840 | SPIMP10g0287130 | Sopen02g024650 |
|  | SHch02g022270 | | SGch02g022190 | Solyc02g079890 |  | Sopen02g024660 |
|  | SHch02g022280 | | SGch02g022200 | Solyc03g007730 |  | Sopen03g031270 |
|  | SHch02g022290 | | SGch02g022210 | Solyc00g154480 |  | Sopen09g010750 |
|  | SHch02g022300 | | SGch02g022220 |  |  |  |
|  | SHch03g006700 | | SGch03g006690 |  |  |  |
|  | SHch05g014260 | | SGch05g015980 |  |  |  |
|  | SHch10g004390 | | SGch09g019180 |  |  |  |
|  |  | | SGch10g004400 |  |  |  |
|  |  | | SGch10g018500 |  |  |  |
| **TPS-c** | SHch09g019240 | | SGch06g031470 | Solyc06g084240 | SPIMP06g0205560 | Sopen06g035610 |
|  |  | | SGch08g004830 | Solyc08g005710 | SPIMP08g0234180 |  |
| **TPS-e/f** | SHch03g005580 | | SGch03g005590 | Solyc03g006550 | SPIMP03g0086110 | Sopen03g002550 |
|  | SHch07g029710 | | SGch07g029580 | Solyc07g066670 | SPIMP07g0233450 | Sopen07g034820 |
|  | SHch08g004730 | | SGch08g004680 | Solyc08g005640 | SPIMP08g0234130 | Sopen08g001700 |
|  | SHch08g004750 | | SGch08g004710 | Solyc08g005665 | SPIMP08g0234160 | Sopen08g001730 |
|  |  | | SGch08g004730 | Solyc08g005670 | SPIMP08g0234190 |  |
|  |  | | SGch08g004760 | Solyc08g005720 | SPIMP08g0234210 |  |
|  |  | | SGch08g004780 |  |  |  |
|  |  | | SGch08g004850 |  |  |  |
| **TPS-g** | SHch05g014270 | | SGch05g015970 | Solyc05g026600 | SPIMP05g0166300 | Sopen05g024370 |
|  | SHch05g017700 | | SGch05g015990 | Solyc10g005390 | SPIMP05g0166310 | Sopen10g001380 |
|  | SHch10g004380 | | SGch10g004390 | Solyc10g005410 | SPIMP10g0287200 | Sopen10g001400 |
|  | SHch10g004400 | | SGch10g004410 |  | SPIMP10g0287210 |  |

Table S4**. TPS genes in the *S. habrochaites* genome**

| Gene Name | Gene ID | Subfamily |
| --- | --- | --- |
| *ShTPS1* | *SHch01g036570* | a |
| *ShTPS2* | *SHch01g036580* | a |
| *ShTPS3* | *SHch01g036590* | a |
| *ShTPS4* | *SHch01g036600* | a |
| *ShTPS5* | *SHch01g040210* | b |
| *ShTPS6* | *SHch01g040220* | b |
| *ShTPS7* | *SHch01g040230* | b |
| *ShTPS8* | *SHch01g040240* | b |
| *ShTPS9* | *SHch01g040250* | b |
| *ShTPS10* | *SHch01g040260* | b |
| *ShTPS11* | *SHch02g018950* | a |
| *ShTPS12* | *SHch02g022220* | b |
| *ShTPS13* | *SHch02g022270* | b |
| *ShTPS14* | *SHch02g022280* | b |
| *ShTPS15* | *SHch02g022290* | b |
| *ShTPS16* | *SHch02g022300* | b |
| *ShTPS17* | *SHch03g005580* | e/f |
| *ShTPS18* | *SHch03g006700* | b |
| *ShTPS19* | *SHch04g009290* | a |
| *ShTPS20* | *SHch04g017410* | a |
| *ShTPS21* | *SHch04g018570* | a |
| *ShTPS22* | *SHch04g019500* | a |
| *ShTPS23* | *SHch05g014260* | b |
| *ShTPS24* | *SHch05g014270* | g |
| *ShTPS25* | *SHch05g017700* | g |
| *ShTPS26* | *SHch06g013250* | a |
| *ShTPS27* | *SHch06g013320* | a |
| *ShTPS28* | *SHch06g016390* | a |
| *ShTPS29* | *SHch06g016400* | a |
| *ShTPS30* | *SHch06g016440* | a |
| *ShTPS31* | *SHch06g016450* | a |
| *ShTPS32* | *SHch06g016510* | a |
| *ShTPS33* | *SHch06g016660* | a |
| *ShTPS34* | *SHch06g031450* | a |
| *ShTPS35* | *SHch07g007840* | a |
| *ShTPS36* | *SHch07g007850* | a |
| *ShTPS37* | *SHch07g007860* | a |
| *ShTPS38* | *SHch07g020130* | a |
| *ShTPS39* | *SHch07g020140* | a |
| *ShTPS40* | *SHch07g020150* | a |
| *ShTPS41* | *SHch07g020570* | a |
| *ShTPS42* | *SHch07g020580* | a |
| *ShTPS43* | *SHch07g020590* | a |
| *ShTPS44* | *SHch07g029710* | e/f |
| *ShTPS45* | *SHch08g004730* | e/f |
| *ShTPS46* | *SHch08g004750* | e/f |
| *ShTPS47* | *SHch08g019920* | a |
| *ShTPS48* | *SHch08g019930* | a |
| *ShTPS49* | *SHch09g019240* | c |
| *ShTPS50* | *SHch09g027610* | a |
| *ShTPS51* | *SHch09g027620* | a |
| *ShTPS52* | *SHch09g027630* | a |
| *ShTPS53* | *SHch10g004380* | g |
| *ShTPS54* | *SHch10g004390* | b |
| *ShTPS55* | *SHch10g004400* | g |
| *ShTPS56* | *SHch12g005560* | a |
| *ShTPS57* | *SHch12g012190* | a |
| *ShTPS58* | *SHun967g006420* | a |
| *ShTPS59* | *SHun967g006430* | a |

Table S5**. RGAs in genomes of five tomato species**

| **RGA family** | ***S. habrochaites*** | ***S. galapagense*** | ***S. lycopersicum*** | ***S. pimpinellifolium*** | ***S. pennellii*** |
| --- | --- | --- | --- | --- | --- |
| CN | 9 | 8 | 31 | 5 | 26 |
| CNL | 36 | 37 | 32 | 44 | 61 |
| NBS | 21 | 22 | 0 | 19 | 45 |
| NL | 58 | 48 | 34 | 39 | 70 |
| TN | 3 | 5 | 5 | 4 | 6 |
| TNL | 26 | 23 | 18 | 23 | 20 |
| TX | 8 | 7 | 13 | 6 | 8 |
| OTHER | 3 | 1 | 1 | 4 | 2 |
| RLK | 517 | 519 | 457 | 396 | 579 |
| RLP | 76 | 71 | 75 | 87 | 81 |
| TM-CC | 153 | 140 | 126 | 159 | 401 |
| **Total** | 910 | 881 | 792 | 786 | 1299 |
